# Editorial: Current Insights Into Complex Post-infection Fatigue Syndromes With Unknown Aetiology: The Case of Myalgic Encephalomyelitis/Chronic Fatigue Syndrome and Beyond

**DOI:** 10.3389/fmed.2022.862953

**Published:** 2022-02-24

**Authors:** Francisco Westermeier, Eliana Mattos Lacerda, Carmen Scheibenbogen, Nuno Sepúlveda

**Affiliations:** ^1^Department of Health Studies, Institute of Biomedical Science, FH Joanneum University of Applied Sciences, Graz, Austria; ^2^Centro Integrativo de Biología y Química Aplicada (CIBQA), Universidad Bernardo O'Higgins, Santiago, Chile; ^3^Department of Clinical Research, Faculty of Infectious and Tropical Diseases, London School of Hygiene and Tropical Medicine, London, United Kingdom; ^4^Charité - Universitätsmedizin Berlin, Freie Universität Berlin, Humboldt Universität zu Berlin and Berlin Institute of Health, Institute of Medical Immunology, Berlin, Germany; ^5^Faculty of Mathematics and Information Science, Warsaw University of Technology, Warsaw, Poland; ^6^CEAUL - Centro de Estatística e Aplicações da Universidade de Lisboa, Lisboa, Portugal

**Keywords:** ME/CFS, Ross River virus (RRV), SARS-CoV-2, long-COVID, post COVID syndrome herpesviruses, enteroviruses, endothelial (dys)function, ICU—rehabilitation

## Introduction

Black plague epidemics in Medieval Europe, the Spanish Flu pandemic during the first world war, and the pandemic of COVID-19 disease are just three devastating examples of the fragile co-existence between human beings and the microbial world. Remarkably, the human immune system with its innate and adaptive arms recognizes and clears the invading pathogens in most cases. However, like a scar after an injury, some people who had suffered from acute infections remain ill long after the clearance of the pathogen itself. These individuals develop complex fatigue-related syndromes whose pathological mechanisms remain poorly understood. A prime example of such syndromes is the Myalgic Encephalomyelitis/Chronic Fatigue Syndrome (ME/CFS) characterized by persistent fatigue and post-exertional malaise among other symptoms (1). Unfortunately, its diagnosis remains challenging due to the inexistence of objective biomarkers that could identify cases. However, researchers are gathering around multidisciplinary networks, such as the US ME/CFS Clinician Coalition and the European Network on ME/CFS, with the aim of fostering collaboration, standardizing research and clinical practices, while accelerating biomarker discovery (2–5). Less-known fatigue-related syndromes have been recently reported after the outbreaks of Ebola virus, Dengue virus, and Chikungunya virus in the Tropics (6–8). However, it is still unclear whether these syndromes constitute clinical entities beyond ME/CFS itself.

**Figure 1:**
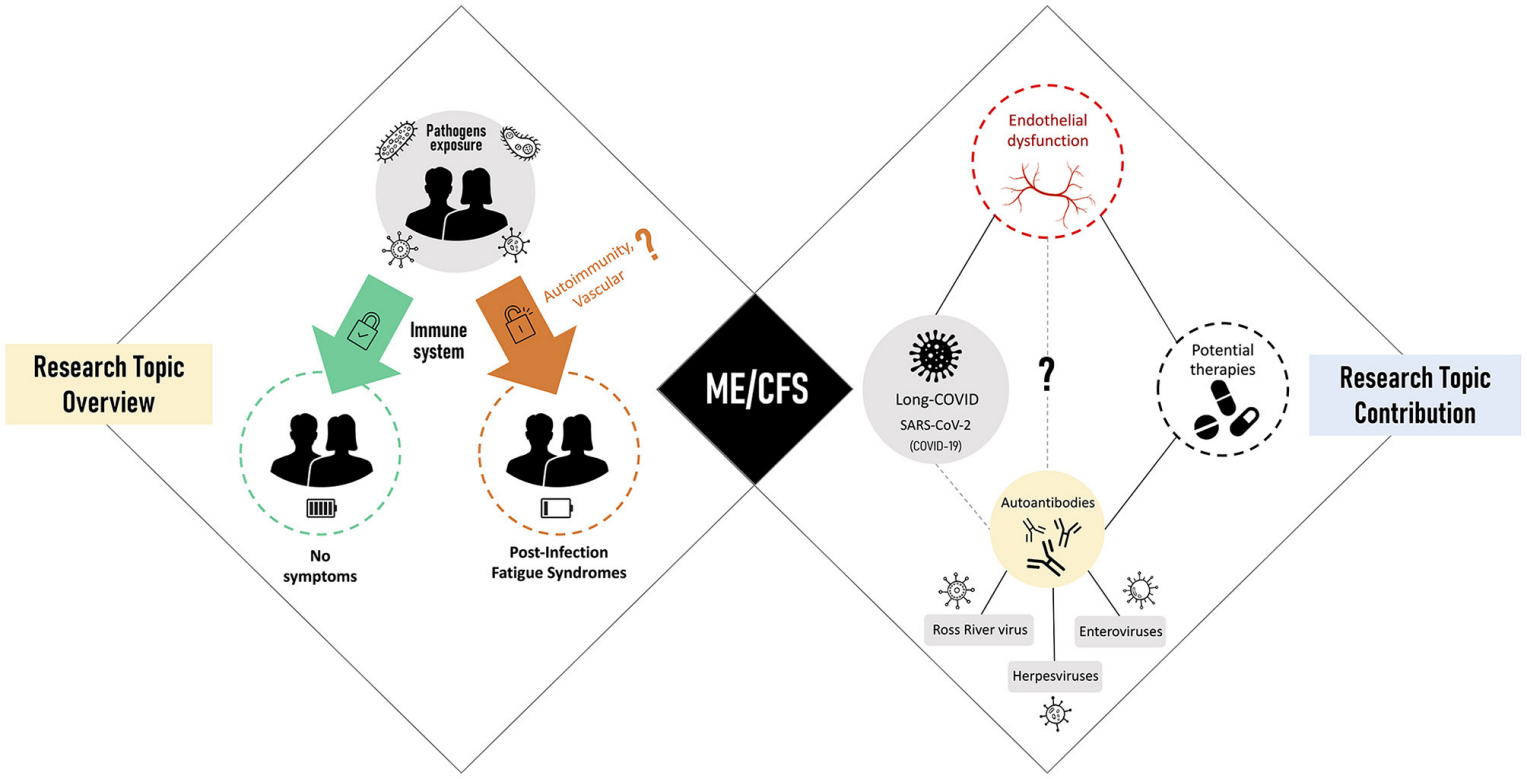
Research Topic overview (left side) linked to post-infectious fatigue syndromes with myalgic encephalomyelitis/chronic fatigue syndrome (ME/CFS) as the main representative (center). Research Topic contribution (right) focused on viral triggers of ME/CFS, disease-related pathomechanisms, and potential therapies.

In this scenario, we invited the research community to contribute with studies on these complex fatigue-related syndromes. Our primary objective was to take the pulse of current data and hypotheses about how these syndromes are initiated and maintained over time. Our second objective was to understand how current insights can lead to successful treatments for patients. With the WHO notification of the COVID-19 as a pandemic on March 11, 2020, our third and final objective was to debate for the first time about ME/CFS as a sequela of post-SARS-CoV-2 infections. The graphical summary of all the contributions received is shown in Figure 1.

## Old and New Viral Triggers of ME/CFS

Early on, it was immediately recognized the impact of herpesviruses on the pathology of ME/CFS (9–11). Follow-up studies made clear that other viruses could also elicit the disease (12). However, the respective pathological mechanisms remain to be uncovered. In this regard, O'Neal and Hanson offered a critical review about past research on enteroviruses as causative agents of ME/CFS. Another interesting review was conducted by Lidbury who discussed the immune evasion strategies of the Ross River virus, which is an arbovirus endemic to Australia, Papua New Guinea, and other islands in the South Pacific. We foresee this review to be useful for understanding post-infection fatigue syndromes due to other arboviruses, such as the Chikungunya, Dengue, and Zika. In this regard, it is a priority to study the burden of post-infection fatigue among Brazilian or Cape Verdean survivors who suffered from recent outbreaks of these arboviruses (13, 14). Finally, Lee et al. and Domingues et al. provided new research on herpesviruses in patients from the United Kingdom ME/CFS biobank. The first study is a rare longitudinal analysis of multiple herpesviruses in patients with ME/CFS; such studies should become standard given the natural fluctuations in disease dynamics. The second study concerns a re-analysis of published serological data using a stratification based on infection and non-infection triggers. The findings of this study clearly show the necessity of stratifying patients adequately, as suggested by Jason et al. (15).

With the onset of the COVID-19 pandemic, a new viral trigger of ME/CFS is currently spreading across the world: SARS-CoV-2. Past experience with the “original” SARS-CoV pandemic suggested this coronavirus as another trigger of ME/CFS (16). Before any mainstream discussion about “long-COVID” or “post-acute sequelae SARS-CoV-2 infection”, Komaroff and Bateman on behalf of the US ME/CFS clinician coalition drafted a sort of memorandum alerting for the devastating long-term consequences in survivors of SARS-CoV-2 infections. In turn, Petracek et al. reported probably the first three ME/CFS cases after 6 months of SARS-CoV-2 infections. Other studies published elsewhere provide further evidence that some long-COVID patients suffer from ME/CFS (17, 18) and, as such, there is a window of opportunity to improve the understanding of both conditions.

## New Perspectives on Disease Pathology and Treatment

A key challenge of investigating ME/CFS is that the disease is likely to be multifactorial and heterogeneous and, therefore, patients might show different pathological pathways that could explain their symptoms. To resolve this, many theoretical papers about possible disease mechanisms emerged in the literature over the years (19–23). In this Research Topic, Stanculescu et al. followed the footsteps of these early theoretical papers by paralleling the pathological mechanisms suggested for patients in an intensive care unit (ICU) and patients with ME/CFS. Their research premise is that the same “vicious circle” between inflammation, oxidative and nitrosative stress, and low thyroid hormone function is operating in both clinical populations. In a follow-up paper, Stanculescu et al. made a comprehensive review of available treatments for ICU patients with the idea of being repurposed to stop that “vicious circle” in patients with ME/CFS. Given the heterogeneous nature of ME/CFS, it is likely that the suggested parallelism might only hold true for some but not all the patients. In another theoretical paper, O'Boyle et al. provided a general discussion about treatment and case management using a previously proposed framework for the natural progression of the disease (24). These authors suggested that pre-disease and early disease call for rehabilitation strategies that could avoid long-term co-morbidity while the management of the established disease should be more holistic and tailored to the specific needs of each patient. The basic question is whether clinicians are able to estimate accurately at which disease stage a patient is.

As a follow-up from early clinical trials in Norwegian patients with ME/CFS (25, 26), Sørland et al. evaluated endothelial function in patients with ME/CFS at baseline and after a therapeutic intervention with cyclophosphamide, an immunosuppressive drug used in cancer. This evaluation was motivated by the growing evidence of vascular abnormalities in ME/CFS (27, 28). The authors also found endothelial dysfunction at baseline, which persisted after treatment irrespective of the clinical response of the patients. Interestingly, the authors also reported a significant correlation between high symmetric dimethylarginine (SDMA) levels and low flow-mediated dilation values. Thus, given that SDMA has been described to reduce the production of nitric oxide (NO) in endothelial cells (29), this study raises a new perspective to address endothelial dysfunction in ME/CFS by combining clinical and metabolic parameters. Endothelial dysfunction and inadequate regulation of blood flow resulting in hypoperfusion of the brain and muscles are considered as key pathological mechanisms in ME/CFS as further outlined in two recent papers (21, 30). There is increasing evidence that autoantibodies directed against vasoregulatory receptors contribute to the vascular dysregulation in ME/CFS (21, 30). These findings open perspectives for therapy. For example, one can target autoreactive B cells or autoantibodies, and preliminary studies provide evidence for clinical efficacy [reviewed in ref. (30)]. The use of drugs that help regulating vascular function is another possibility to treat patients with ME/CFS.

## Conclusions

In conclusion, this Research Topic collects further pieces of evidence about how various viruses including SARS-CoV-2 can trigger ME/CFS. The neglect of research in ME/CFS during the last decades has left patients, carers, and clinicians alike adrift without a licensed drug to use in the disease. On the one hand, the COVID-19 pandemic will result in an unprecedented explosion of ME/CFS cases. At the same time, this pandemic is the perfect storm that can motivate different stakeholders, including funders and clinicians, to take the necessary steps to accelerate research on ME/CFS and other post-infectious syndromes. If taken, these steps will bring hope to all those outstanding patients who have been homebound or even bedridden for many years but neglected by national health authorities.

## Author Contributions

All authors contributed to this editorial and approved the final version.

## Funding

FW received funding from ME Research UK (SCIO charity number SC036942). EL received funding from the National Institutes of Health (ref. NIH 2R01AI103629), and the ME Association (UKMEB–ME Association, Grant PF8947_ME Association). CS received funding from the Weidenhammer Zoebele Foundation, Germany. NS received funding from Fundação para a Ciência e Tecnologia, Portugal (ref. UIDB/00006/2020), and the Polish National Agency for Academic Exchange, Poland (ref. PPN/ULM/2020/1/00069/U/00001).

## Conflict of Interest

The authors declare that the research was conducted in the absence of any commercial or financial relationships that could be construed as a potential conflict of interest.

## Publisher's Note

All claims expressed in this article are solely those of the authors and do not necessarily represent those of their affiliated organizations, or those of the publisher, the editors and the reviewers. Any product that may be evaluated in this article, or claim that may be made by its manufacturer, is not guaranteed or endorsed by the publisher.
